# Clinical results of endoscopic sciatic nerve decompression for deep gluteal syndrome: mean 2-year follow-up

**DOI:** 10.1186/s12891-016-1062-3

**Published:** 2016-05-20

**Authors:** Myung-sik Park, Sun-Jung Yoon, Sung-yeop Jung, Seung-Ho Kim

**Affiliations:** Department of Orthopedic Surgery, Research Institute of Clinical Medicine of Chonbuk National University- Biomedical Research Institute of Chonbuk National University Hospital, Jeonju, South Korea

**Keywords:** Endoscopic sciatic nerve decompression, Sciatic nerve entrapment, Deep gluteal syndrome

## Abstract

**Background:**

The purpose of this study is to assess the effectiveness of endoscopic sciatic nerve decompression and evaluated the differences of clinical results between atraumatic and traumatic groups.

**Methods:**

Sixty consecutive patients. We retrospectively reviewed sixty consecutive patients without major trauma (45 hips) or with major trauma (15 hips) groups to compare the outcomes of endoscopic treatment.). The mean follow-up period was 24 ± 2.6 months (range, 24–38.4 months).

**Results:**

The mean duration of symptoms was 14.1 months (range, 12 to 32 months). Compromising structures were piriformis muscle, fibrovascular bundles, and adhesion with scar tissues. The mean VAS score for pain decreased from 7.4 ± 1.5 to 2.6 ± 1.5 (*P* = .001). The mean mHHS increased from 81.7 ± 9.6 to 91.8 ± 7.6 (*P* = .003). Clinically, positive paresthesia and seated piriformis test were statistically significant to diagnosis sciatic entrapment syndrome. Paresthesia and sitting pain were significantly improved at the final follow-up (*P* = .002). More favorable outcome was observed a group without major trauma. No complication was observed.

**Conclusions:**

Endoscopic sciatic nerve decompression is a safe and effective procedure for the management of DGS. Patients with major trauma could have poor clinical outcome. Seated piriformis test, FADIR, and tenderness of sciatic notch are maybe useful guide for pre and postoperative evaluation of DGS.

## Background

**Fig. 1:**
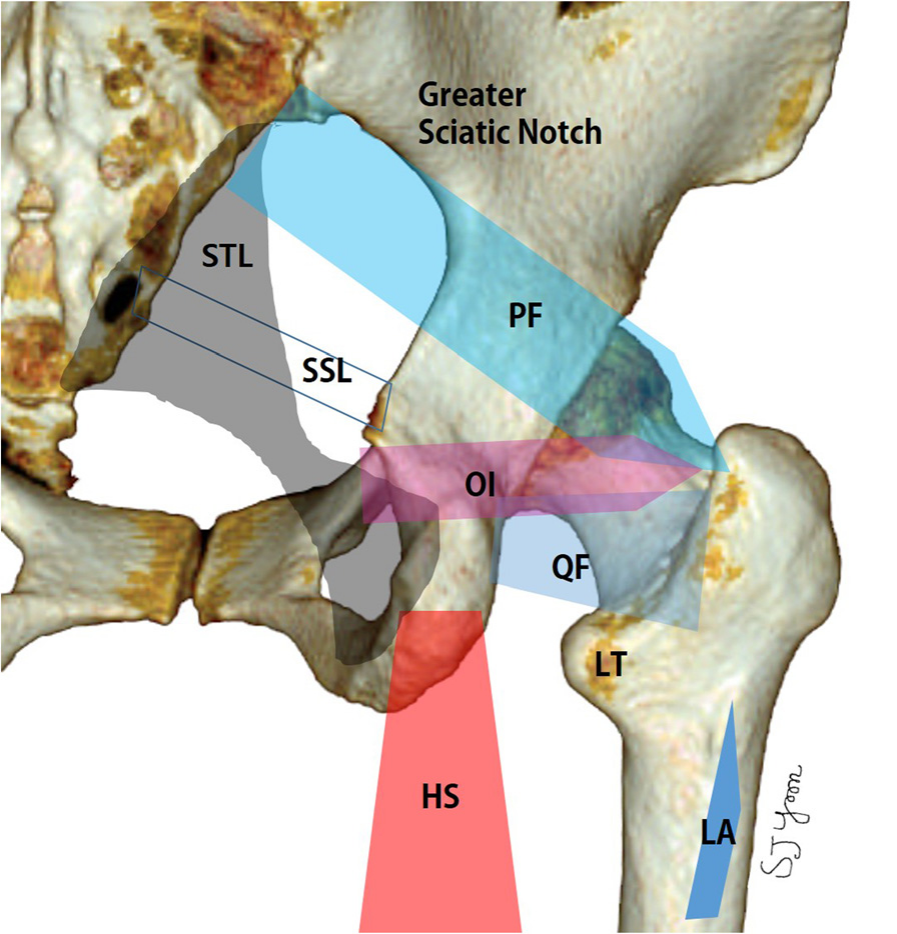
Schematic image of the deep gluteal space, HS, hamstring origin; LA, linea aspera; LT, lesser trochanter; OI, obturator internus; PF, piriformis; SSL, sacrospinous ligament; QF, Quadratus Femoris

Deep gluteal syndrome (DGS) involves pain in the buttock caused from entrapment of the sciatic nerve in deep gluteal space [1]. The boundaries of deep gluteal space are femoral neck anteriorly, gluteus maximus posteriorly, linea aspera of proximal femur laterally, sacrotuberous ligament medially, inferior margin of the sciatic notch superiorly and hamstring muscle inferiorly (Fig. 1). The space contains piriformis, obturator internus/externus, gemelli, quadratus femoris, hamstring, gluteal nerves and lateral ascending vessels of the medial femoral circumflex artery. Any contents of deep gluteal space can cause sciatic nerve entrapment syndrome [2]. Posttraumatic DGS can be occurred by scarring around the piriformis muscle and the sciatic nerve as well as the adherence of the nerve to the posterior pelvic coulum [3].

Patients presenting with sciatic nerve entrapment often complain symptoms of sitting pain (inability to sit for 30 min), walking pain, radicular pain of the lower back or hip, and paresthesia of the affected buttock and inguinal area [4, 5]. Diagnosis can made by several physical examinations include tenderness on sciatic notch, Flexion-ADduction-Internal Rotation (FADIR) test, Pace sign [6], Lasègue test and seated piriformis test [2, 4, 7]. A variant of the Freiberg test involves flexion, adduction, and internal rotation of the hip [3, 8]. The Pace sign is pain and weakness with resisted abduction and external rotation of the hip [6]. The Lasègue sign is pain with straight leg raise testing (to 90° hip flexion) [9, 10]. Usually, magnetic resonance arthrography (MRA) and electromyography (EMG) usually don’t provide proper information for DGS. So, clinical symptoms and physical examinations are essential for diagnosis of sciatic nerve entrapment syndrome [2]. In the patients of DGS who have moderate to severe symptoms or undergo failure of conservative treatment, sciatic nerve decompression through open or endoscopic technique can be performed for release of pain [2, 4, 7].

There are difficulties to diagnose by one of clinical presentations, physical examinations, or objective evaluations. Moreover, it still remains unclear which symptoms or signs of this complex syndrome would be resolved after endoscopic decompression. To our knowledge, this is the first study regarding differences between atraumatic and traumatic deep gluteal syndrome.

The purpose of this study is to assess 1) which clinical presentations or physical examinations will improve after the endoscopic sciatic nerve decompression 2) the effectiveness of this endoscopic procedure, 3) differences of clinical outcome between groups associated with or without major trauma.

## Methods

**Fig. 2:**
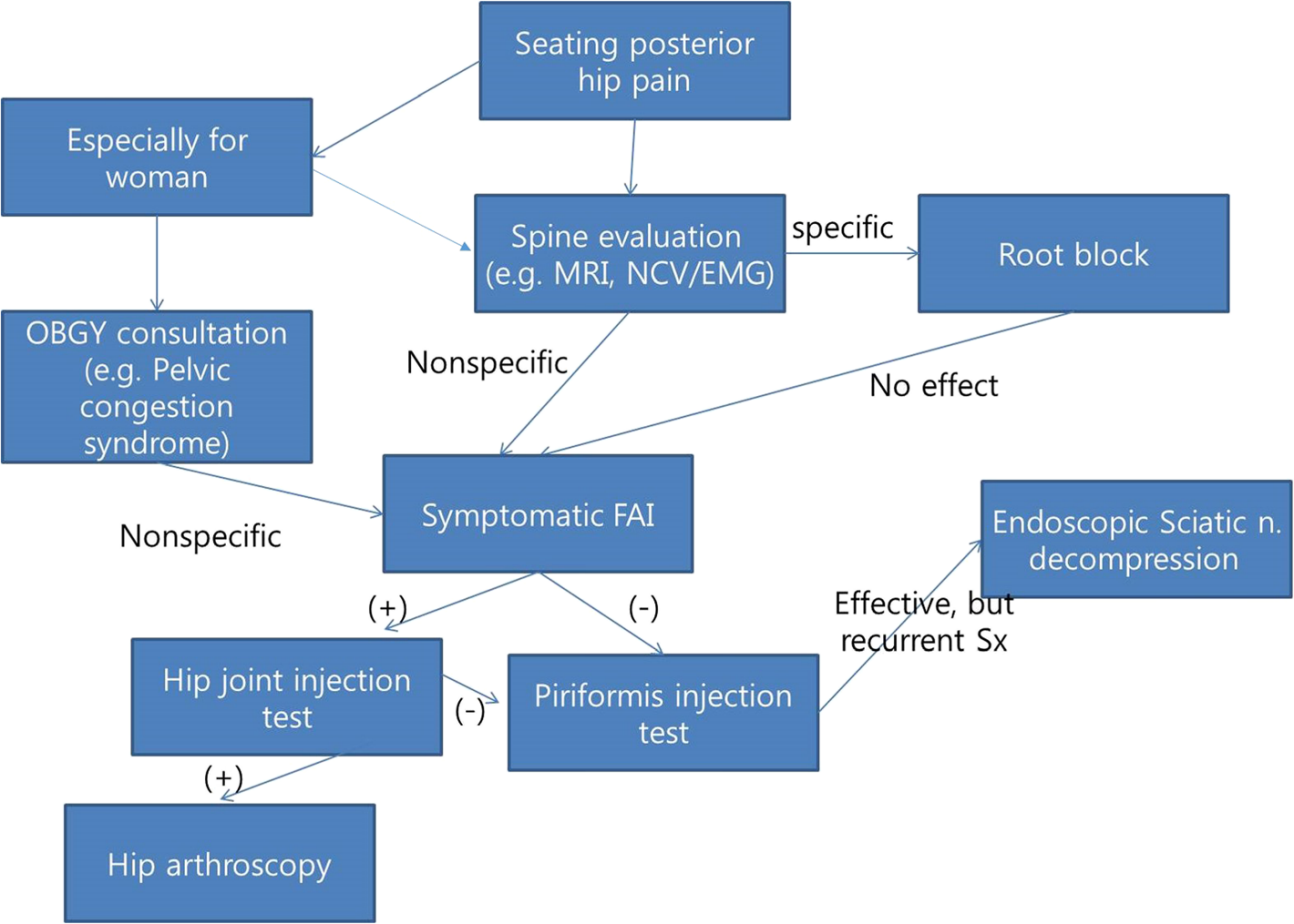
Flow-chart showing treatment process. A flow-chart showing the decision making process for endoscopic sciatic nerve decompression of seating posterior hip pain

Endoscopic sciatic nerve release was performed from September 2009 to June 2013. This study was approved by institutional review board of the Chonbuk National University Hospital. Total of 66 patients who had endoscopic sciatic nerve decompression for sciatic nerve entrapment syndrome with a minimum 24 months follows up were included in this study. Three patients excluded, who had taken total hip arthroplasty, two patients suffered from direct nerve injury after acetabular fracture and one patient with severe spondylosis. The rest 60 patients were included in this study. There were 33 males and 27 females with a mean age of 48.60 ± 11.46 years (range, 20–70 years). The mean follow-up period was 24 ± 2.6 months (range, 24–38.4 months). The mean duration of symptoms was 10.6 ± 0.9 months (range, 4–30 months). A comprehensive back and hip physical examination ruled out the spine disease and sacroiliac joint disease. Our treatment protocol is shown in Fig. 2.

The clinical diagnosis of the DGS has been advocated by patient history, clinical presentations and physical examinations. We applied described clinical symptoms and physical examinations to elicit the posterior pain according to Martin et al [2]. Clinical presentations included 1) walking pain, 2) sitting pain (in ability to sit for >30 min), 3) radicular pain and 4) paresthesia. The physical examinations included 1) tenderness on sciatic notch, 2) FADIR test, 3) Pace sign, 4) Lasègue test and 5) seated piriformis test. A test was considered positive withthe recreation of the posterior pain [8].

The anteroposterior pelvic radiograph and MRA were made with the patient supine on the table with both lower extremities oriented in 20° of internal rotation using a boots in order to maximize the length of the femoral neck. All radiographic images were acquired digitally through a Picture Archiving and Communication System (PACS, Marotech, Maroview, Seoul).

The radiologic measurements such as the neck-shaft angle (NSA), lateral center-edge (LCE) angle on pelvis AP radiographs and femoral, acetabular anteversion and ischiofemoral space (IF space) on a MRA were assessed for all patients [11, 12]. The measuring of the ischiofemoral space is the smallest distance between the lateral cortex of the ischial tuberosity and medial cortex of the lesser trochanter, and quadratus femoris space. All MRA examinations were evaluated for qualitative change by consensus by one musculoskeletal radiologist and two orthopedic surgery fellows. We also check the electromyography for the symptoms of radicular pain in eight patients selectively to differentiate other compressive neuropathies of sciatic nerve.

**Fig. 3:**
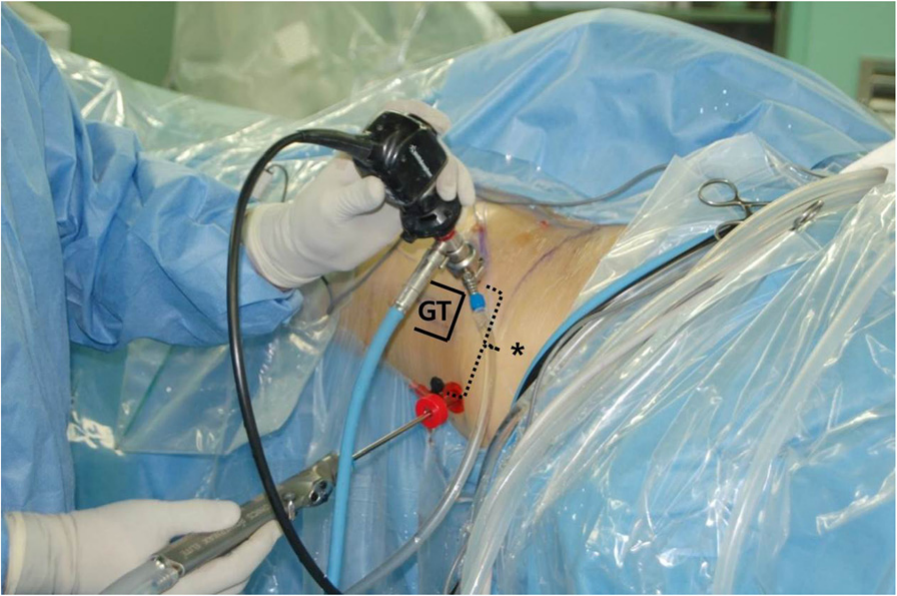
Endoscopic portals for sciatic nerve decompression. The anterolateral portal placement is one cm anterolateral to the anterior corner of the greater trochanter (GT). The posterolateral portal is four-fingerbreadth posterior to the anterior portal*

Surgical procedure was done with a supine position on the hip arthroscopic table. Diagnostic hip arthroscopy was performed for all patients with deep gluteal syndrome. If the patient had definitive intraarticular lesion under hip arthroscopy, the lesion would be tried to treat especially if their posterior hip pain has alleviated by intra-articular hip injection test. Then the sciatic nerve was explored. For approach to the deep gluteal area, we make a posterolateral and/or axillary portals. The axillary portal is placed 3–4 cm superior or inferior to center between anterolateral and posterolateral portals. Through the endoscopy, sciatic nerve was detected, that passes around piriformis, obturator internus and gemelli muscles in deep gluteal space (Fig. 3). Sciatic nerve release was performed by removal of fibrovascular scar bands or splitting tendinous portion of piriformis, obturator internus, and quadriceps femoris muscles and or bony spicules which compromised sciatic nerve excursion [2]. The fibrovascular scars were delicately cauterized by use of a radiofrequency probe and removed by use of arthroscopic shaver or dissection scissors. After release, using nerve retractor, we identified improvement of tightness and gliding of the sciatic nerve while internal and external rotation or flexion and extension of hip repeatedly [13]. Postoperative one day, we allowed partial weight bearing with crutch and full weight bearing at three weeks.

Clinical outcomes was assessed by persistence or not of the symptoms and physical examinations, VAS score, modified Harris Hip Score (mHHS) [14], Hip outcomes score (HOS daily life), SF-12 (12-Item Short Form Survey) and modified Benson surgical outcomes rating [3] which divided as poor, fair, good, and excellent (Table 1). To compare the outcomes of endoscopic sciatic nerve decompression between fracture and non-fracture patients, we divided two groups as like who had not taken any pelvic or acetabular surgery group (45 patients) and associated prior pelvic or acetabular fracture surgery group, 15 patients. Of these patients, acetabular fracture in 11 patients and associated with pelvic ring fracture in four patient.

**Table 1:** Benson surgical outcomes rating

Outcomes	Symptoms
Excellent	No pain with prolonged periods of sitting (>30 min), strenuous activity, bending, twisting, stairs, rapid walking, jogging.
Good	No pain with short periods of sitting (≤30 min) or daily activities or mild pain with prolonged periods of sitting or strenuous activity.
Fair	Occasional mild pain with short periods of sitting or normal daily activities or moderate pain with prolonged sitting or strenuous activity.
Poor	Severe pain with short periods of sitting or normal daily activities, little change from preoperative level of pain associated with sciatic nerve.

Statistical analyses were performed using a SPSS (version 8.0). Categorical variables are reported as frequencies and percentage and compared between preoperative and postoperative results with Fisher’s exact tests. Continuous variables are summarized as means and SDs and compared between groups with independent-samples *t* tests. All statistical test were 2-sided, and *P* values < .05 were considered statistically significant.

## Results

On plain pelvis, preoperative NSA was 131.9 ± 4.7°, and LCE angle was 29.8 ± 5.4°. Using with a MRA femoral anteversion 15.9 ± 9.9°, acetabular anteversion 13.8 ± 5.6° and ischiofemoral space was 13.4 ± 3.8 mm in lesion site and 15.1 ± 4.4 mm in contralateral side (*P* = .000) was measured (Table 2). Ninety percent of patient complaining sit pain was improved after surgery. Preoperative symptoms of 1) sit pain (inability to sit for more than 30 min), 2) paresthesia, 3) radicular pain, and 4) walking pain were improved 90.9 % (*P* = .002), 88.8 %, 45.3 %, and, 50 % respectively at 2 year follow-up. Preoperative sitting pain and paresthesia were significantly improved after endoscopic decompression.

**Table 2:** Clinical presentations and physical examinations

Clinical presentations	Preop	Postop	*P*
Walking pain	11 (18.3 %)	6 (10 %)	0.240
Sit pain (inability to sit for more than 30 min)	53 (88.3 %)	5 (8.3 %)	0.000
Radicular pain	12 (20 %)	6 (10 %)	0.130
Paresthesia	36 (60 %)	4 (6.7 %)	0.000
Physical exam			
Tenderness	42 (70 %)	14 (23.3 %)	0.000
FADIR	26 (43.3 %)	9 (15 %)	0.000
Pace’sign	14 (23.3 %)	5 (8.3 %)	0.060
Lasègue	10 (16.7 %)	3 (5.0 %)	0.070
Seated piriformis	43 (71.7 %)	4 (6.7 %)	0.000

For physical examination, 90 % of patients showing positive seated piriformis test was disappeared of sign after surgery. Preoperative positive physical examination of 1) tenderness on sciatic notch, 2) FADIR test 3) Pace sign, 4) Lasègue test, and 5) seated piriformis test were improved 90.7 %, 70.2 %, 66.6 %, 65.3 %, and 64.3 %, respectively at two year follow-up. Preoperative sciatic notch pain, FADIR test, and Seated piriformis test were significantly improved after sciatic nerve decompression (*P* < .001). Postoperative clinical symptoms improvement and positive physical examination were shown Table 2.

During surgery, we found the operative findings compatible with femoroacetabular impingements in 27 hips included 7 hips as a mixed type, 12 hips as a cam type, and 8 hips as a pincer type. Arthroscopic FAI surgery was underwent for only symptomatic FAI (16 of 27 patients) proven by preoperative evaluation including intra-articular injection test.

**Fig. 4:**
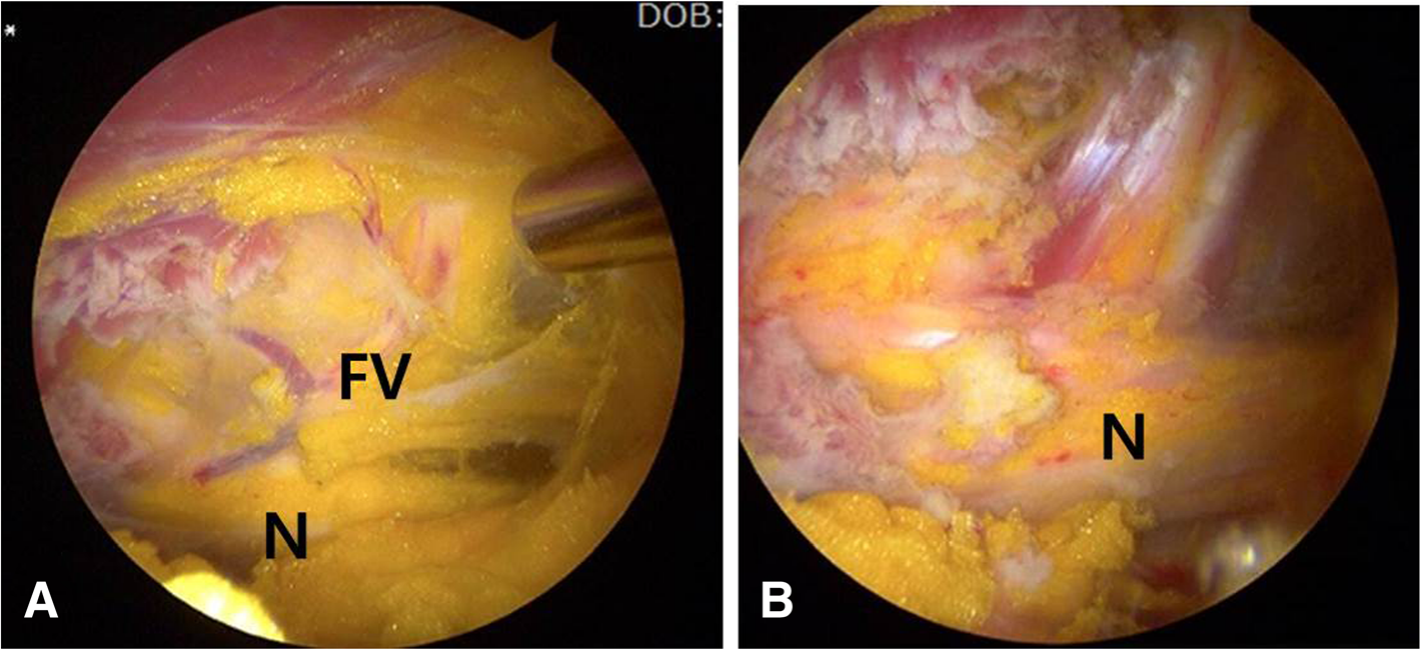
Sciatic nerve entrapment by fibrovascular bands. **a** Endoscopic view of sciatic nerve (N) by fibrovascular bands (FV). **b** Endoscopic view of sciatic nerve (N) after decompression

In deep gluteal space, as anatomical etiologies of sciatic nerve entrapment, the fibrovascular scar bands were observed in 27 hips (Table 3), variation of the tendinous portion of piriformis muscle in 25 hips, variation of the obturator internus in four hips, hypertrophy of the quadratus femoris muscles in three hips and thickened bursa extending from greater trochanter in three hips (Fig. 4), heterotopic ossification around fracture site in one hips, and simple fibrous band dissection in seven hips.

**Table 3:** Caused structures of sciatic nerve entrapment

Compromising structures	Number of hips (*n* = 60)
Fibrous scar bands with Gluteus maximus bursa (3)	27
Piriformis muscle and triceps coxae	25
with Obturator internus tendon (4) with Quadratus femoris muscles (3)	
Vascular compression	7
Heterotopic ossification	1

The mean of ischiofemoral space in DGS groups are 13.6 ± 3.8 mm (range, 4.1–21.4) and contralateral side are 15.2 ± 4.5 mm (range, 4.1–26.9) (*p* = 0.000). And other radiologic markers were described (Table 4).

**Table 4:** Results of imaging study of DGS patients

Variables	Mean value		*P*
Neck-shaft angle (°)	131.9 ± 4.7	2 (>140°)	3 (<125°)
Femoral anteversion (°)	15.9 ± 9.9	12 (20°)	14 (<10°)
LCE^a^ angle (°)	29.8 ± 5.4	1 (>42°)	15 (<26°)
	Ipsilateral side	Contralateral side	
Ischiofemoral space (mm)	13.4 ± 3.80	15.1 ± 4.40	.000

^a^ *LCE* Lateral center edge angle

Postoperatively, patient satisfaction was evaluated as excellent in 15, good in 38, and fair in seven patients. There were no postoperative complications as like compartment syndrome or sciatic nerve injuries. Patient satisfaction in trauma group was statistically lower than it in disease group.

## Discussion

Deep gluteal syndrome is a condition in which the sciatic nerve is compressed by any structures in deep gluteal space. The chief symptom were caused by sciatic nerve entrapment surrounding structures which usually causes buttock pain and a complained of the inability to sit for a long period of time. Pain became worse during sitting, walking and flexion movement combined with internal rotation of the hip. And also patients may complain radicular pain of affected leg, much like nerve root pain associated with lumbar disc disease [4]. Previously as the term “piriformis syndrome” that implies a type of deep gluteal syndrome [15]. However, any structures in deep gluteal region can cause this syndrome by compression sciatic nerve, so sciatic nerve entrapment syndrome or deep gluteal syndrome may be a proper representation [16]. There are a number of etiological factors of sciatic nerve entrapment syndrome, such as direct trauma of buttock or pelvis, hypertrophy of muscles in deep gluteal region, hematoma or neoplasm and anatomical variants between piriformis and sciatic nerve, etc [17–19]. Be aware of possible anatomical variation bifurcation of the sciatic nerve through the muscles. In this study, fibrous scar band and anatomical variation of tendinous portion of the piriformis were most frequent intraoperative findings. Otherwise, intraneural and perineural fibrosis was the most common arthroscopic finding in major trauma group. The prevalence of the piriformis and sciatic nerve anomaly in piriformis syndrome patients has been reported by others [20]. It was not significantly different from what is thought to be a normal population, it indicated that this anomaly may not be as important in the pathogenesis of piriformis syndrome as previously thought.

In this study, all patients were evaluated with physical examinations, routine pelvis radiography, and also MRA. We couldn’t find a specific feature of DGS in radiologic variables such as, neck-shaft angle, femoral anteversion and acetabular anteversion in this study. However, by previous study, in some positions that cause sciatic nerve compression, such as the FADIR test, electromyography may have greater specificity and sensitivity than other available tests for the diagnosis of piriformis syndrome [7]. Bryan et al suggested layered concepts of anatomical structure of the hip. The pathologies of osseous and cartilage layers can affected the clinical presentations of musculotendinous or neural layers [18]. In this study, we repaired symptomatic labral lesions and corrected FAI deformity [21].

A high index of suspicion is needed to make this diagnosis of DGS in a patient with posterior hip or leg pain in absence of lumbar pathology. This pain is usually aggravated while sitting with more than 30 min or no back pain. Sciatic nerve entrapment due to varicose vein or pyomyositis of piriformis muscle in a pediatric patient was also reported [22, 23]. We suggested repeated trauma of the piriformis muscle as sudden stretching of muscle or long-term exercise and traumatic subgluteal muscular damages. These surrounding soft tissue structures and heterotopic ossification sometimes may be act as pressure to the passage of the sciatic nerve into the piriformis muscle and decompression is the last resort.

In this study, tenderness of deep gluteal space, FADIR test, and seated piriformis test showed more statistical significance (*P* < .001) to diagnosis DGS, but Pace sign (*P* = .06) and Lasègue test has no significance (*P* = .070).

According to Martin et al., MRI usually doesn’t provide sufficient information for DGS [2, 24]. In our study, of 60 patients with sciatic nerve entrapment syndrome, only six patients showed minor swelling of sciatic nerve. Decreased distance of ischiofemoral space have a statistical significance with symptom of DGS (*P* < 0.001), which might be related with ischiofemoral impingement. The diagnostic efficacy of MR neurography has been reported by others [25]. Piriformis muscle asymmetry and sciatic nerve hyperintensity at the sciatic notch exhibited a 93 % specificity and 64 % sensitivity. Cutoff values of ischiofemoral and quadratus femoris space has been propose by Torriani et al. ≤ 17 mm, and ≤ 8 mm, subsequently.

Sciatic nerve neuropathy associated fracture or reconstructive surgery of the acetabulum were managed by open method according to Issack et al [5]. After the open decompression, most of sensory symptoms are improved for minimum of one year, but there were no patients with worsening of neurologic examination after release. They reported also improved symptoms of a tenderness and or paresthesia of affected leg after surgery and some improvement of foot drop. However, there are risks associated with open technique such as hematoma, infection, poor cosmesis, and long rehabilitation time [3]. This endoscopic management shows improvement of sitting pain and sensory changes around buttocks and no complications.

This study has several limitations, including a retrospective cohort study, small numbers of case and relatively short follow-up duration. And few patients in this study had endoscopic surgery for sciatic nerve entrapment syndrome and FAI at the same time. For proper evaluation of endoscopic surgery for sciatic nerve entrapment syndrome, additional well-designed prospective studies, in a group of patients with only sciatic nerve entrapment syndrome without intraarticular lesions are necessary.

## Conclusion

Endoscopic sciatic nerve decompression is a safe and effective procedure for the management of DGS. Patients with major trauma could have poor clinical outcome. Seated piriformis test, FADIR, and tenderness of sciatic notch are maybe useful guide for pre and postoperative evaluation of DGS.

## Abbreviations

DGS: deep gluteal syndrome
EMG: electromyography
FADIR: Flexion-ADduction-Internal Rotation
FAI: femoroacetabular impingement
HOS: hip outcome score
IF: ischiofemoral
LCE: lateral center-edge angle
mHHS: modified Harris hip score
MRA: magnetic resonance arthrography
NSA: neck-shaft angle
VAS: visual analogue scale
